# Prediction of Cavitation Evolution and Cavitation Erosion on Centrifugal Pump Blades by the DCM-RNG Method

**DOI:** 10.1155/2021/6498451

**Published:** 2021-11-15

**Authors:** Han Zhu, Ning Qiu, Chuan Wang, Qiaorui Si, Jie Wu, Fanjie Deng, Xiang Liu

**Affiliations:** ^1^National Research Center of Pumps, Jiangsu University, Zhenjiang, 212013 Jiangsu, China; ^2^Hainan Vocational University of Science and Technology, Haikou, 571126 Hainan, China; ^3^College of Hydraulic Science and Engineering, Yangzhou University, Yangzhou, 225009 Jiangsu, China

## Abstract

Cavitation can reduce the efficiency and service life of the centrifugal pumps, and a long-term operation under cavitation conditions will cause cavitation damage on the surface of material. The external characteristic test of the IS65-50-174 single-stage centrifugal pump was carried out. Moreover, the cavitation mechanism under specific conditions was analyzed by numerical simulation. Considering the macroscopic cavitation flow structure in the centrifugal pump, three different cavitation erosion prediction methods were used to predict the erodible areas. The results show that the calculation results obtained by the density correction method (DCM) can well match the flow characteristics of the centrifugal pump under the rated conditions. When the centrifugal pump head drops by 3%, cavitation mainly occurs on the suction surface, and the cavity on the pressure surface is mainly concentrated near the front cover. The cavitation prediction method based on the time derivation of pressure change is not suitable for centrifugal pumps, while the prediction result of the erosive power method is more reasonable than the others. At time 0.493114 s, the maximum erosive power appears on the blade near the volute tongue, and its value is 1.46*e* − 04 W.

## 1. Introduction

**Figure 1:**
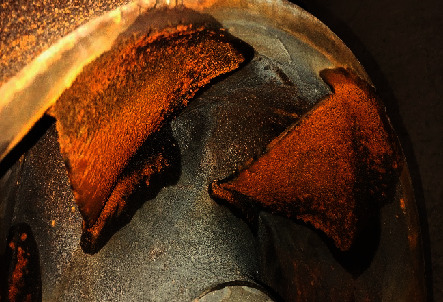
Cavitation erosion in the centrifugal pump.

Cavitation is a common phenomenon in centrifugal pumps. When the centrifugal pump is running, the liquid pressure in local areas will be lower than the saturated vapor pressure at the operation temperature, resulting in cavitation [1]. The area of blade surface where cavitation occurs will be damaged under the repeated action of impact load, as shown in Figure 1. At the same time, it will also cause increased noise and severe vibration of the centrifugal pump [2, 3]. Therefore, it is of great significance to carry out cavitation erosion research on centrifugal pumps [4–6].

So far, researchers from various countries have studied the cavitation erosion by experimental and numerical methods [7–10]. Dular et al. [11] used PIV and PLIF technology to measure the internal cavitation flow field of the centrifugal pump and obtained the transient and time-averaged velocity field and vapor volume fraction distribution around the blade. Bilus and Predin [12] studied the effect of installing a rectifier on the inlet pipe on the cavitation performance of the centrifugal pump through experiments, and the use of the rectifier improved the cavitation state. Bachert et al. [13] used PIV testing technology combined with high-speed cameras to study cloud-like cavitation at the volute tongue of centrifugal pumps and explored the reasons for the deterioration of centrifugal pump performance under high flow rate conditions. Wang et al. [14] studied the cavitation at the inlet of the centrifugal pump impeller and initially obtained the relationship between the cavitation distribution in the impeller and the vibration and noise characteristics of the pump. Fu et al. [15] used high-speed photography and pressure pulsation measurement technology to systematically analyze the low-frequency cavitation-induced pulsation characteristics of centrifugal pumps under low flow rate conditions.

It is challenging to use experimental methods to study cavitation and the unsteady evolution of cavitation in complex hydraulic machinery. At the same time, there are also difficulties in accurately measuring the velocity flow field of the reentrant flow, and capturing the vortex structure of the shedding cavity. Compared with the experiment, more flow field details can be obtained by numerical simulation, offering a better explaining of the cavitation mechanism. Based on the three-component continuity equation of liquid phase, vapor phase, and noncondensable gas, Hong [16] constructed a nonlinear cavitation model and calculated the cavitation flow field in the axial flow pump. Caridad et al. [17] used numerical simulation to study the cavitation phenomenon of centrifugal pumps under unsteady-state conditions. The accumulation of cavitation caused blockage of the flow channel and increased hydraulic loss. Through numerical simulation, Tan et al. [18] found that under unsteady conditions, the internal cavitation of the centrifugal pump impeller has less effect on the flow pattern under bean flow rate than low flow rate influence. Wang et al. [19] established the RZGB cavitation model considering the rotation effect, the structural characteristics of large curvature, and the compressibility of the liquid when studying the cavitation flow field in the centrifugal pump.

Based on the microjet method, Peters et al. [20] made a prediction of cavitation erosion on a NACA0009 hydrofoil. Wang [21] proposed a cavitation prediction method based on *Matlab* image processing of cavitation clouds in the centrifugal pump. Their results show that the cavitation location in the centrifugal pump is basically the same as the cavitation erosion area. Li et al. [22] conducted a comparative analysis of the erodible area on the NACA0015 hydrofoil based on the first-order deviation of pressure versus time and proved that this method can better match the erodible area on the hydrofoil. Through experiments, Qiu et al. [23] compared the cavitation erosion of NACA0015 hydrofoils with different leading-edge structures and conducted a quantitative analysis of the cavitation impact energy. Usta et al. [24] compared the differences between the Intensity Function Method, Gray level Method, and Erosive Power Method. Then, they compared and predicted the erosion area on a propeller. The results show that the EPM method has advantages in predicting propeller cavitation erosion.

In this paper, the density-corrected RNG *k* − *ε* model was used for cavitation simulation, and three different cavitation erosion prediction methods were used to predict cavitation erosion on the blade surface of the centrifugal pump.

## 2. Centrifugal Pump Parameters and Experimental Setup

In this paper, a single-stage centrifugal pump of type IS65-50-174 was studied. The main parameters of this test pump are shown in Table 1.

**Figure 2:**
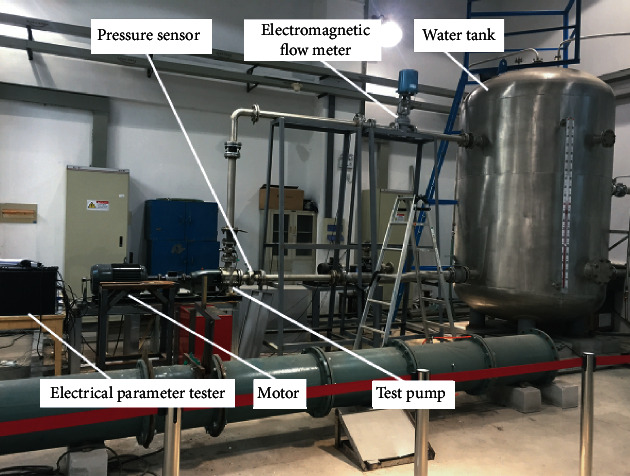
Cavitation test rig.

The experiments were finished at the Research Center of Fluid Machinery Engineering Technology of Jiangsu University, and the system is shown in Figure 2. The system is mainly composed of two parts: water circulation circuit and data acquisition system.

## 3. Numerical Simulation Methods

### 3.1. Continuity Equation and Momentum Equation

In the simulation, the vapor-liquid two-phase flow is generally assumed to be homogeneous. The Navier-Stokes equation based on the Newtonian fluid was used in this simulation. The equation is formulated in the Cartesian coordinate system as

(1) $$\begin{array}{c} \frac{\partial \rho_{m}}{\partial t}+\frac{\partial \left(\rho_{m}u_{j}\right)}{\partial x_{j}}=0,\\ \frac{\partial \left(\rho_{m}u_{i}\right)}{\partial t}+\frac{\partial \left(\rho_{m}u_{i}u_{j}\right)}{\partial x_{j}}=-\frac{\partial p}{\partial x_{i}}+\frac{\partial }{\partial x_{j}}\times \left[\left(\mu_{m}+\mu_{T}\right)\left(\frac{\partial u_{i}}{\partial x_{j}}+\frac{\partial u_{j}}{\partial x_{i}}-\frac{2}{3}\frac{\partial u_{i}}{\partial x_{j}}\delta_{ij}\right)\right],\\ \frac{\partial \rho_{l}\alpha_{l}}{\partial t}+\frac{\partial \left(\rho_{l}\alpha_{l}u_{j}\right)}{\partial x_{j}}=m^{+}+m^{-},\\ \rho_{m}=\rho_{l}\alpha_{l}+\rho_{v}\alpha_{v},\\ \mu_{m}=\mu_{l}\alpha_{l}+\mu_{v}\alpha_{v}, \end{array}$$

where the subscript *i*, *j* indicates the coordinate direction, *u* indicates the velocity, *p* indicates the pressure, *ρ*_*l*_ indicates the liquid density, *ρ*_*v*_ indicates the vapor density, *α*_*v*_ indicates the vapor volume fraction, *α*_*l*_ indicates the liquid volume fraction, *μ*_*l*_ indicates the liquid laminar viscosity, *μ*_*v*_ indicates the vapor laminar viscosity, *μ*_*T*_ indicates the turbulent viscosity, *m*^+^ indicates the vapor condensation rate, and *m*^−^ indicates the vapor evaporation rate. *ρ*_*m*_ denotes vapor-liquid mixed-phase density, *μ*_*m*_ denotes vapor-liquid mixed-phase laminar viscosity, *t* indicates the time, *x*_*i*_, *x*_*j*_ are the coordinates in the *i* and *j* directions, and *δ*_*ij*_ indicates the shear stress.

### 3.2. Cavitation Model

The Zwart model [25, 26] is a cavitation model based on the mass transport equation, which describes the cavitation phase change process mainly by establishing the transport relationship between the vapor and liquid phases. Its evaporation rate *m*^+^ and condensation rate *m*^−^ are defined as follows:

(2) $$\begin{array}{c} m^{+}=C_{\text{dest}}\frac{3\alpha_{\text{nuc}}\left(1-\alpha_{v}\right)\rho_{v}}{R_{B}}{\left(\frac{2}{3}\frac{p_{v}-p}{\rho_{l}}\right)}^{1/2},\;p<p_{v},\\ m^{-}=-C_{\text{prod}}\frac{3\alpha_{v}\rho_{v}}{R_{B}}{\left(\frac{2}{3}\frac{p-p_{v}}{\rho_{l}}\right)}^{1/2},\;p>p_{v}. \end{array}$$

In the formula, *R*_*B*_ is the bubble radius, *α*_nuc_ is the volume fraction of gas nuclei, *p*_*v*_ is the saturated vapor pressure, *C*_prod_ is the rates of steam condensation when the local static pressure is greater than the saturation steam pressure, and *C*_dest_ is the rate of steam evaporation when the local static pressure is lower than the saturated vapor pressure. The values of the coefficients in the model are [25] *R*_*B*_ = 1 × 10^−6^*m*, *C*_prod_ = 0.01, *C*_dest_ = 50, and *α*_nuc_ = 1 × 10^−4^.

### 3.3. Turbulence Model

This simulation was based on the RNG *k* − *ε* turbulence model. To more accurately simulate the development of cavitation in the centrifugal pump, the compressibility of the mixing of the vapor and liquid phases was considered, and the mixed density was corrected by the density corrected method (DCM). Turbulent viscosity is defined as follows:

(3) $$\begin{array}{c} f_{\text{DCM}}=\rho_{v}+{\left(\frac{\rho_{m}-\rho_{v}}{\rho_{l}-\rho_{v}}\right)}^{N}\cdot \left(\rho_{l}-\rho_{v}\right),\\ \mu_{\text{T}‐\text{DCM}}=\frac{C_{\mu }k^{2}}{\varepsilon }f_{\text{DCM}}, \end{array}$$

where *N* is taken as 10 according to literature recommendations [27].

### 3.4. Numerical Setup and Grid Validation

**Figure 3:**
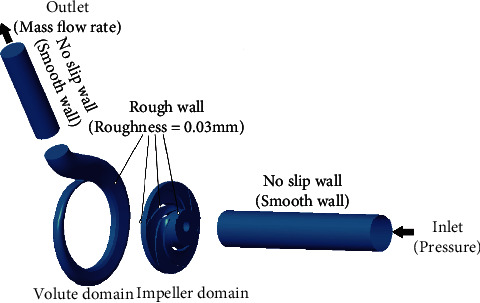
The calculation domain.

Water temperature of experiment and simulation was maintained at about 23.5°C.  Figure 3 shows the calculation domain and boundary conditions. In order to avoid the inlet and outlet reflux of the calculation domain, an inlet expansion pipe was set before the impeller calculation domain, and an outlet expansion pipe was set after the volute calculation domain. The walls of the extension were all set as nonslip walls. The inlet was set as a pressure inlet with a value of 1 atm. Moreover, the outlet was set as a mass flow rate outlet with a value of 50 m^3^/h. The wall surface roughness of the impeller and volute was set to 0.03 mm. Furthermore, the impeller was set as a rotating domain with a speed of 2900 rpm, and the other parts were set as the static domains.

**Figure 4:**
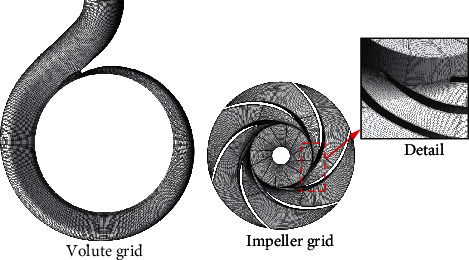
The detail of grid.

The calculation model was divided into hexahedral structure using *ANSYS ICEM* software. In order to reduce the interference of the grid quality on the simulated performance of the centrifugal pump, the near-wall area of the blade was meshed densely to better control the grid structure of the boundary. The grid details are shown in Figure 4. Considering the influence of the grid on the calculation, the RNG *k* − *ε* turbulence model was used to analyze the grid independence of the computational domain.  Table 2 shows the grid information of the computational domain. The change of head is used as the criterion for judging the applicability of the grid.

(4) $$\begin{array}{c} \text{Head}=\frac{P_{\text{out}}-P_{\text{in}}}{\rho g}. \end{array}$$

**Figure 5:**
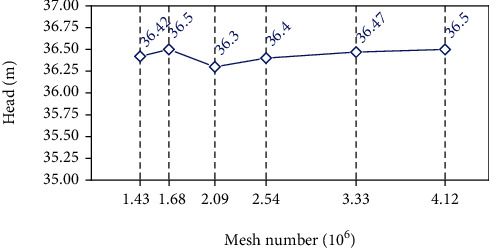
The head under different mesh number.

**Figure 6:**
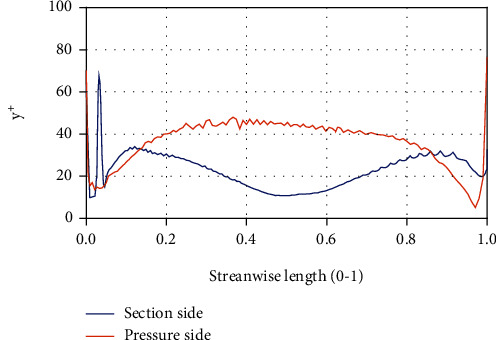
*y* ^+^ on the blades.

Figure 5 shows the head changes under different grid conditions. It can be seen that as the number of grids increases, the head tends to be stable. Considering the computing duration and resources, final option 4 with a mesh number of 2538949 was selected for calculation. Under this scheme, the *y*^+^ value of the near-wall surface of the blade is within 100, which meets the calculation requirements of the RNG *k* − *ε* turbulence model, as shown in Figure 6.

## 4. Results and Discussion

### 4.1. Experimental Verification and Steady Analysis

Under the conditions of rated flow and speed, the simulated head is 36.39 m and the experimental result is 35.71 m. The relative error is 2%, indicating that the simulation has high reliability.

**Figure 7:**
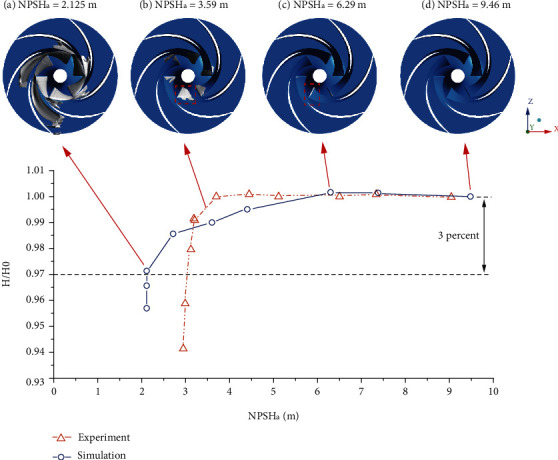
Cavitation performance curve. (vapor volume fraction is used for showing the cavitation evolution; isosurface = 0.1)

Figure 7 shows the cavitation performance curve of the test pump. At the rated flow rate, the inlet pressure of the centrifugal pump was reduced by adjusting the vacuum tank. With the pressure gradually reduced, a set of heads and net positive suction heads were obtained, and the cavitation performance curve can be obtained. The net positive suction head calculation formula is

(5) $$\begin{array}{c} {\text{NPSH}}_{a}=\frac{p_{s}}{\rho g}+\frac{v_{s}^{2}}{2g}-\frac{p_{v}}{\rho g}. \end{array}$$

In engineering, it is generally believed that when the head drops by 3%, the cavitation in the pump is in a critical state, and the performance of the centrifugal pump drops sharply at this state. It can be seen from Figure 7 that the experimental results show that the net positive suction head is about 3 m when the head drops by 3%, and the simulation result is about 2.12 m. The critical NPSH_*a*_ obtained by simulation is slightly smaller than the experimental result. The reason may be that the simulation calculation is in an ideal working state with less interference. Figures 7(a)–7(d) show the simulated cavitation distribution under different NPSH_*a*_. In the initial stage, when NPSH_*a*_ = 9.46 m, there is no cavitation in the centrifugal pump. As the inlet pressure decreases, the NPSH_*a*_ drops to 6.29 m. A slender cavitation zone attached to the suction surface appears on the inlet side of the blade. When the pressure is further reduced, the covered area of the cavity attached to the blade becomes larger, which affects the stability of the flow in the pump to a certain extent, resulting in a drop in the head of the centrifugal pump, as shown in Figure 7(c). At this state, the head is about 99% of the noncavitation head. During the evolution of cavitation, the cavity will gradually become larger and thicker. When NPSH_*a*_ = 2.125 m, the centrifugal pump head is 3% lower than the noncavitation, as can be seen in Figure 7(d). At this state, the cavitation area fills the entire flow channel and the blocking directly leads to a sharp drop in pump performance.

### 4.2. Analysis of the Unsteady Cavitation Performance

An unsteady simulation of the internal flow field when NPSH_*a*_ = 2.125 m under the rated flow was performed. The total duration of unsteady calculation was set to 0.4138 s (the time required for 20 cycles of the impeller rotation), and the time step was set to 0.00022989 s (the case calculated after each 1/90 cycle of impeller rotation). One to ten times iteration was chosen to make the calculation reach the convergence accuracy. The convergence accuracy was set to 0.0001 to ensure the reliability of unsteady calculations. After the impeller rotated 20°, a result file was saved.

**Figure 8:**
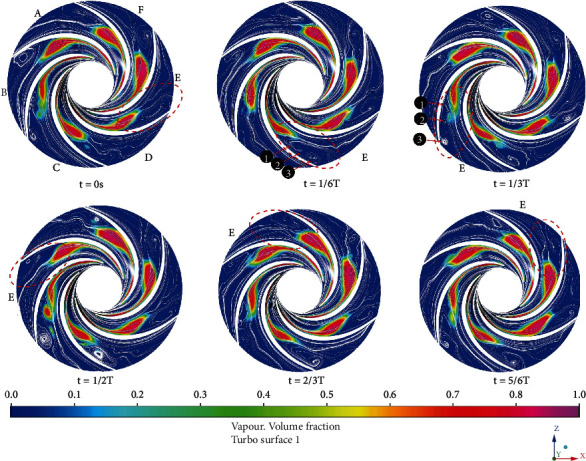
The streamline of water superficial velocity and the vapor volume fraction on span = 0.5 turbo surface.

Figure 8 shows the liquid phase streamlines and cavitation volume fraction at span = 0.5 during one cycle of the impeller rotation. The streamline near the pressure surface of the blade is neat, but the streamline near the suction surface has some vortexes, as shown in the red dashed area in the figure. The evolution of the vortexes presents obvious unsteady characteristics. A large recirculation zone appears near the exit of the flow channel E. When the time progresses from 2/6 T to 3/6 T, the three vortexes in the flow channel E merge one again and continue to grow over time, while the center of the vortex moves to the outlet of the flow channel. The existence of vortexes reflects the instability of the flow. And the instability of flow is an important reason that affects the performance of the centrifugal pump, especially the drop in head and efficiency. It can be seen from the figure that there is an obvious boundary between the vortex structure and the cavitation structure, and the vortex zone appears behind the vapor phase region. Cavitation occurs on the suction surface of the blade and develops backward. It is attached to the blades and gradually becomes longer and thicker. At the same time, the attached cavity will roll away from the blade in the closed area of the cavitation at the tail and begin to block the flow path due to the rotation of the impeller. The existence of cavities changes the flow filed, and a velocity vortex is formed behind the cavities.

**Figure 9:**
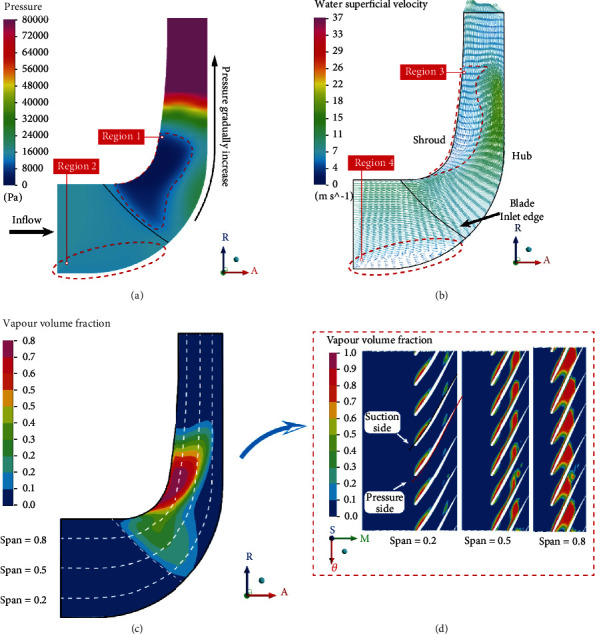
The mean distribution on the impeller shaft surface. (a) Pressure. (b) Velocity. (c) Vapor volume fraction.

Figure 9 shows the mean value distribution of each parameter in the impeller shaft surface at a typical time *t* = 3/6 T. It can be seen from Figures 9(a) and 9(b) that water enters from the impeller inlet and the pressure is low. With the rotation of the blades, the pressure in the flow channel shows an overall upward trend and exceeds 80,000 Pa at the impeller outlet. The fluid velocity near the hub is relatively low, maintaining between 0 and 7 m/s. A certain degree of backflow exists, resulting in lower pressure in this area than in other areas of the impeller inlet, as shown in region 2. There is irregular low-pressure region 1 near the front cover, and cavitation will mainly occur in this area, as shown in Figure 9(c). At the same time, it can be seen that at a position closer to the outlet, a certain range of low-speed area appears. Some vortexes appear in the middle and downstream of the impeller flow channel. Due to the unstable flow, the average velocity in this area is low. It can be seen from Figure 9(c) that the cavitation is irregularly distributed and is basically consistent with the shape of the low-pressure zone. The most severe area of cavitation is located near the front cover, and the cavitation volume fraction gradually decreases from the front cover to the rear cover. There is only a small amount of cavitation at the root of the blade near the rear cover.

Figure 9(d) shows the expanded view of the cavitation volume fraction on the blade at this moment. It can be seen from the figure that on the cross section of span = 0.2, cavitation is mainly distributed on the suction surface of the blade, in the form of attached cavitation. On the cross-section of span =0.5, the attached cavities on the suction surface become longer in the chord length direction of the blade and shell at the closure of the attached cavities. A small area of cavitation appears on the pressure side of the blade inlet. On the cross-section of span = 0.8, the cavitation on the pressure side of the blade is severe. And then, cavitation on the pressure side and cavitation on the suction side gather together, completely blocking the flow channel.

**Figure 10:**
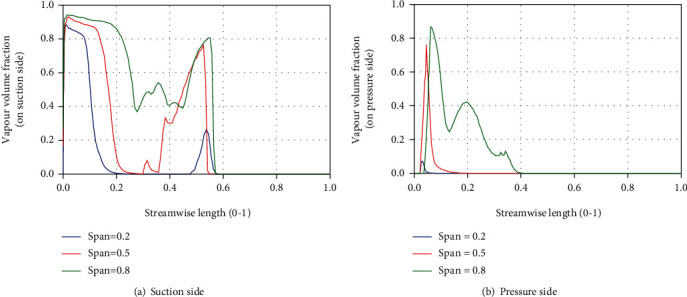
Distribution of cavitation volume fraction on a typical blade (*t* = 3/6 T).

Figure 10 shows the volume fraction distribution of cavitation on different spans of a typical blade. Comparing Figures 10(a) and 10(b), it can be seen that in the same span, the cavitation coverage length of the suction surface is greater than that of the pressure surface, indicating that cavitation mainly occurs on the suction surface of the blade. It can be seen from Figure 10(a) that the cavitation volume fraction on the span = 0.8 is greater than span =0.5 and 0.2 when streamwise length is between 0 and 0.4. The maximum cavitation volume fraction is 0.94 at span = 0.8, the maximum cavitation volume fraction is 0.93 at span = 0.5, and the maximum cavitation volume fraction is 0.89 at span = 0.2. The three maximum values all appear near the inlet edge of the blade. As the streamline length moves from the inlet to the outlet, the cavitation volume fraction gradually decreases and then increases again. The secondary peak of the cavitation volume fraction at span = 0.8 is 0.81, and the secondary peak at span = 0.5 is 0.76, and the secondary peak at span = 0.2 is only 0.26. It can be seen from Figure 10(b) that the cavitation volume fraction on the pressure surface span = 0.8 is above zero with a streamwise length between 0 and 0.4, and on the span = 0.5, it is streamwise distributed between 0 and 0.2; and the streamwise distribution is between 0 and 0.07 at span = 0.2. These indicate that on the pressure surface, cavitation is more likely to occur near the front cover.

### 4.3. Cavitation Erosion Prediction on Centrifugal Pump Blades

**Figure 11:**
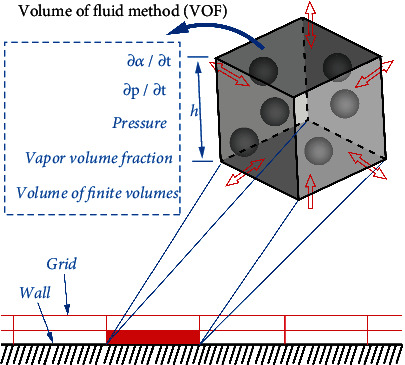
The simulation method based on VOF.

**Figure 12:**
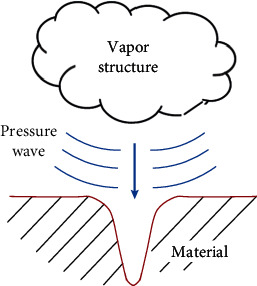
The occurrence of cavitation erosion.

It is based on the simulation method of VOF (Figure 11) to predict cavitation erosion. Cavitation erosion is a microscopic and transient process, but it is also affected by macroscopic flows. From the energy point of view, the collapse of the cavitation bubble will produce a pressure pulse. This pressure wave is one of the factors that cause cavitation damage, as shown in Figure 12. Pressure waves can be generated by the collapse. Based on the hypothesis of Qiu et al. [23, 28–32], the potential energy of the cavity structure is defined as

(6) $$\begin{array}{c} E_{\text{pot}}=\Delta p\times V_{\text{vap}},\\ \Delta p=\left(p-p_{\text{sat}}\right). \end{array}$$

According to the definition of potential energy, the erosive power function can be constructed. The cavitation volume is reduced when cavity collapses and the pressure wave is released. The erosive power function *p*_pot_ can be defined as

(7) $$\begin{array}{c} p_{\text{pot}}=\frac{\partial E_{\text{pot}}}{\partial t}=\Delta p\times \frac{\partial V_{\text{vap}}}{\partial t}+V_{\text{vap}}\times \frac{\partial \Delta p}{\partial t}=\left(p-p_{\text{sat}}\right)\times V_{\text{cell}}\times \frac{\partial \alpha }{\partial t}+V_{\text{cell}}\times \alpha \times \frac{\partial p}{\partial t},\;\text{if }P_{\text{pot}}\geq \varepsilon_{\text{pot}}, \end{array}$$

(8) $$\begin{array}{c} \alpha =\frac{V_{\text{vap}}}{V_{\text{cell}}}=\frac{\rho -\rho_{l}}{\rho_{v}-\rho_{l}}. \end{array}$$

Li et al. [22] used the method of pressure change rate, and the cavitation intensity function *I*_pressure_ is defined as

(9) $$\begin{array}{c} I_{\text{pressure}}=\frac{dp}{dt},\;\text{if }I_{\text{pressure}}\geq \varepsilon_{\text{pressure}}. \end{array}$$

Dular et al. [11] proposed a method for predicting cavitation erosion based on the volume of the cavity. The cavitation erosion function *I*_vapor_ is defined as

(10) $$\begin{array}{c} I_{\text{vapor}}=\frac{{dV}_{v}}{dt}\left(p-p_{v}\right),\;\text{if }I_{\text{vapor}}\geq \varepsilon_{\text{vapor}}. \end{array}$$

In the formula, *p* is the pressure surrounding the vapor (Pa); *p*_sat_ is the saturated steam pressure (Pa); *V*_vap_ is the vapor volume (m^3^); *V*_cell_ is the volume of the grid (m^3^); *α* is vapor volume fraction; and *ε*_pressure_, *ε*_pot_, and *ε*_vapor_ are threshold.

**Figure 13:**
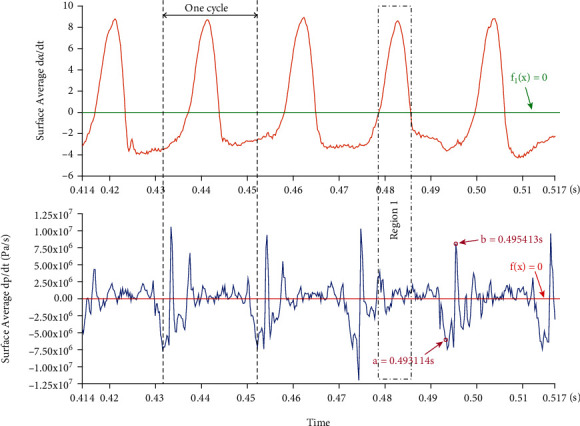
Time evolution of erosion parameters on a typical blade.

**Figure 14:**
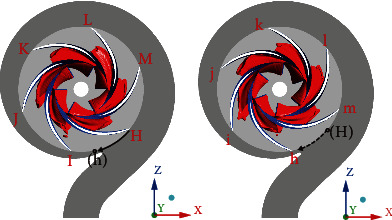
Location of blades at typical times.

Figure 13 shows the time evolution of erosion parameters on a typical blade. It can be seen from the figure that the values of *surface-averageddp*/*dt* and *surface-averagedda*/*dt* present obvious periodic characteristics, and the time of each cycle is about 0.0207 s, which is consistent with the time of one revolution of the impeller. This phenomenon reflects the unsteady characteristics of the cavitation in the pump. With the rotation of the impeller, the cavitation in the pump changes continuously. *Surface-averagedda*/*dt* fluctuates around *f*_1_(*x*) = 0. When the value is greater than 0, the cavitation coverage area near the blade becomes larger. Similarly, *surface-averageddp*/*dt* fluctuates around *f*(*x*) = 0. When the value is less than 0, it indicates that at this moment, the covered cavity has caused the average surface pressure to decrease. The fluctuation of *surface-averageddp*/*dt* is small when the value of *surface-averagedda*/*dt* is above 0, as shown in region 1 in the figure. When the *surface-averagedda*/*dt* is less than 0, the cavitation coverage area decreases, and the *surface-averageddp*/*dt* appears maximum and minimum values in a single cycle. In order to better predict the cavitation erosion situation in the pump, as shown in Figure 14, two typical moments *a* = 0.493114 s and *b* = 0.495413 s are selected for analysis. The selected typical blade is located at the H position at time *a* and h position at time *b*.

**Figure 15:**
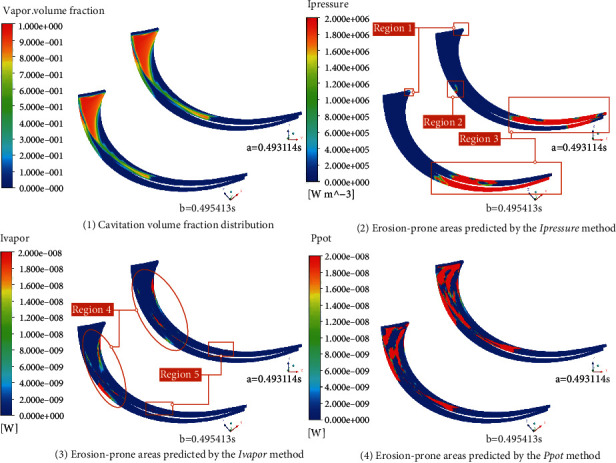
Cavitation erosion prediction results on a typical blade.

**Figure 16:**
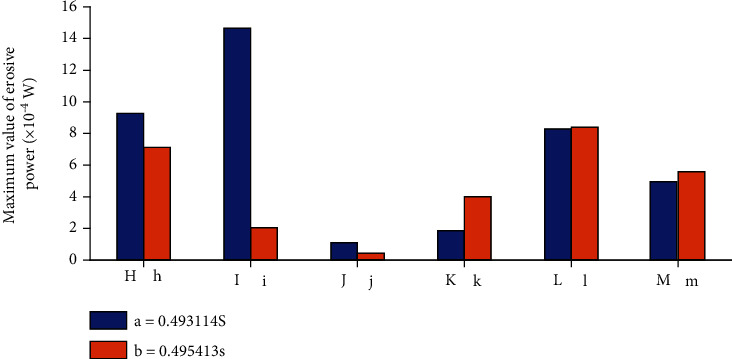
Maximum erosive power on blades.

Based on the simulation results, three methods are used to predict the cavitation erosion distribution on a typical blade, and the thresholds are set to 0. The result is shown in Figure 15. It can be seen from the figure that the erodible area predicted by *I*_pressure_ is in the middle and downstream of the blade (shown in region 3 in the figure), and there is no cavitation coverage in this area. At the same time, there is a small erodible area at the entrance of the blade (shown in region 1 in the figure). Compared with Figure 15(1), it is found that there is also an erodible area near the cavitation closing line of the pressure surface (shown in region 2 in the figure). The erosion area predicted by the *I*_pressure_ method is quite different: the erosion area in the centrifugal pump obtained from the experiment mainly exists in the part covered by the cavitation. Therefore, this method is not suitable for predicting the cavitation erosion area in the centrifugal pump. Figure 15(3) shows the cavitation erosion results obtained by using the *I*_vapor_ prediction method. From the figure, it can be seen that at time *a* and time *b*, the erodible area predicted by this method is smaller than by the other two methods. Compared with Figure 15(1), the erosion area is mainly concentrated near the vapor-liquid boundary line, which is more obvious near the boundary between the covers and the blade (region 4 in the figure). There are some point-like erodible areas near the cavitation closing line (as shown in region 5 in the figure). The trend of the erodible area predicted by this method is basically consistent with the results obtained by other scholars, but it cannot capture the erosion in the cavitation coverage area. Figure 15(4) shows the erosion area predicted by using the erosive power method. It can be observed from the figure that the erosion area develops from the blade inlet to the blade outlet, and the area covered by cavitation shows erosion, which is consistent with the trend of the experimental results of Wang et al. [19, 21]. Compared with the other two methods, this method has advantages in predicting the erodible area in the centrifugal pump. The erosive power method can also be used to quantitatively analyze the erosive power on different blades.  Figure 16 shows the statistical results of the maximum erosive power on the blade at different positions at times *a* and *b*.

It can be observed from Figure 16 that, when the blade rotates from time *a* to time *b*, the erosive power of the blades at positions H, I, and J becomes smaller, while the erosive power at positions K, L, and M becomes larger. At time *a*, the maximum value of erosive power on the blades appears on the blade at position I, and its value is 1.46*e* − 04 W. The minimum value appears on the blade at position J, and its value is 1.10*e* − 05 W. At time *b*, after the blades have rotated 40°, the maximum value of the blades appears at position l, and the minimum value of 4.40*e* − 06 W appears on the blade at position j. At time *b*, the blade at position j rotated by the blade at position J at time *a*. This shows that, after being rotated to 40°, the minimum value still appears on the same blade. Consider the *dp*/*dt* term in formula (7):

(11) $$\begin{array}{c} \frac{dp}{dt}=\frac{p_{t}-p_{t-i}}{i}. \end{array}$$

In the formula, *i* = 0.00022989 s represents the time interval of one step of simulation calculation.

When the blade passes near the tongue, due to the sudden change of the flow channel, the pressure near this position has suddenly increased. Therefore, it is predicted that there is a higher value of erosive power near the position I at time *a*. At the next moment, the flow path gradually becomes larger, and there is no longer a sudden increase in pressure. Therefore, the predicted erosive power is lower.

## 5. Conclusion

The unsteady cavitation in the centrifugal pump is studied and analyzed by the method of combining experiment and numerical simulation, and the results show that

1. the calculated results of the DCM method with a compressibility correction of the turbulence model match with the experimental results well, especially under rated conditions
2. the existence of cavitation in the centrifugal pump causes the blockage of the flow channel and the generation of vortexes, which leads to the decline of the performance of the centrifugal pump
3. when the head of the centrifugal pump drops by 3%, cavitation is mainly concentrated on the blades near the front cover
4. the pressure derivative and volume fraction derivative on the typical blade show a periodic change. The change period is 0.0207 s, which is consistent with the rotation cycle of the impeller
5. comparing the three cavitation erosion prediction methods, it is found that the cavitation erosion prediction method based on the rate of pressure change is not suitable for centrifugal pumps, and the erosion area predicted by the erosive power method is more reasonable. At *a* = 0.493114 s, the maximum erosive power appears on the blade near the separating tongue, and its value is 1.46*e* − 04 W

In a future work, it is necessary to set a cavitation erosion experiment. The simulated and experimental results will be compared, and the pulse energy by cavitation will be quantified. These would provide more theoretical support for engineering application.

## Figures and Tables

**Table 1:** The main parameters of test pump.

Parameter	Value
Design flow rate (*Q*_*d*_)	50 m^3^/h
Design head rise (*H*_*d*_)	34 m
Rotational speed (*n*)	2900 rpm
Number of blades	6
Specific speed	88.6
Outlet width of impeller (*d*)	12 mm
Outlet pipe diameter of pump (*D*_*d*_)	65 mm
Inlet pipe diameter (*D*_*s*_)	74 mm
Impeller diameter (*D*_2_)	174 mm

**Table 2:** The information of mesh.

	Grid number
Mesh 1	1429074
Mesh 2	1683736
Mesh 3	2087120
Mesh 4	2538949
Mesh 5	3331173
Mesh 6	4114547

## Data Availability

The data that support the findings of this study are available from the corresponding author upon reasonable request.

## References

[1] Franc J. P., Michel J. M. (2004). *Fundamentals of Cavitation, vol. 76*.

[2] Agostino L., Salvetti M. V. (2007). *Fluid Dynamics of Cavitation and Cavitating Turbopumps*.

[3] Pan S., Peng X. (2013). *Physical Mechanism of Cavitation*.

[4] Chen Z. X., Hu H. X., Guo X. M., Zheng Y. G. (2022). Effect of groove depth on the slurry erosion of V-shaped grooved surfaces. *Wear*.

[5] Zhou W., Qiu N., Wang L., Gao B., Liu D. (2018). Dynamic analysis of a planar multi-stage centrifugal pump rotor system based on a novel coupled model. *Journal of Sound and Vibration*.

[6] Wang H., Long B., Wang C., Han C., Li L. (2020). Effects of the impeller blade with a slot structure on the centrifugal pump performance. *Energies*.

[7] Song Q. N., Tong Y., Li H. L. (2021). Corrosion and cavitation erosion resistance enhancement of cast Ni-Al bronze by laser surface melting. *Journal of Iron and Steel Research International*.

[8] Li Z. X., Zhang L. M., Ma A. L. (2021). Comparative study on the cavitation erosion behavior of two different rolling surfaces on 304 stainless steel. *Tribology International*.

[9] Zhang L. M., Li Z. X., Hu J. X. (2021). Understanding the roles of deformation-induced martensite of 304 stainless steel in different stages of cavitation erosion. *Tribology International*.

[10] Shi L., Zhu J., Tang F., Wang C. (2020). Multi-Disciplinary optimization design of axial-flow pump impellers based on the approximation model. *Energies*.

[11] Dular M., Bachert R., Stoffel B., Širok B. (2005). Experimental evaluation of numerical simulation of cavitating flow around hydrofoil. *European Journal of Mechanics-B/Fluids*.

[12] Bilus I., Predin A. (2009). Numerical and experimental approach to cavitation surge obstruction in water pump. *International Journal of Numerical Methods for Heat &Fluid Flow*.

[13] Bachert R., Stoffel B., Dular M. (2010). Unsteady cavitation at the tongue of the volute of a centrifugal pump. *Journal of Fluids Engineering*.

[14] Wang Y., Liu H., Yuan S., Tan M., Wang K. (2012). Experimental test on cavitation vibration and noise of centrifugal pumps under non-design conditions. *Journal of Agricultural Engineering*.

[15] Fu Y., Yuan J., Yuan S. (2015). Numerical and experimental analysis of flow phenomena in a centrifugal pump operating under low flow rates. *Journal of Fluids Engineering*.

[16] Hong F. (2016). *Application of Nonlinear Cavitation Model in Numerical Calculation of Hydraulic Machinery Cavitation, [Ph.D. thesis]*.

[17] Caridad J., Asuaje M., Kenyery F., Tremante A., Aguillón O. (2008). Characterization of a centrifugal pump impeller under two-phase flow conditions. *Journal of Petroleum Science and Engineering*.

[18] Tan L., Zhu B., Cao S., Wang Y., Wang B. (2014). Numerical simulation of unsteady cavitation flow in a centrifugal pump at off-design conditions. *Proceedings of the Institution of Mechanical Engineers, Part C: Journal of mechanical engineering science*.

[19] Wang J., Wang Y., Liu H., Si Q., Dular M. (2018). Rotating corrected based cavitation model for a centrifugal pump. *Journal of Fluids Engineering*.

[20] Peters A., Sagar H., Lantermann U., el Moctar O. (2015). Numerical Modelling and Prediction of Cavitation Erosion. *Wear*.

[21] Wang J. (2015). *Numerical Simulation and Experimental Tests for Cavitation and Induced Erosion in Hydraulic Apparatus*.

[22] Li Z., Pourquie M., Van Terwisga T. (2014). Assessment of cavitation erosion with a URANS method. *Journal of Fluids Engineering*.

[23] Qiu N., Zhou W., Che B., Wu D., Wang L., Zhu H. (2020). Effects of microvortex generators on cavitation erosion by changing periodic shedding into new structures. *Physics of Fluids*.

[24] Usta O., Korkut E. (2019). Prediction of cavitation development and cavitation erosion on hydrofoils and propellers by Detached Eddy Simulation. *Ocean Engineering*.

[25] Zwart P., Gerber A., Belamri T. A two-phase flow model for predicting cavitation dynamics.

[26] Wang H., Qian Z., Zhang D., Wang T., Wang C. (2020). Numerical study of the normal impinging water jet at different impinging height, based on Wray–Agarwal turbulence model. *Energies*.

[27] Huang B., Wang G., ZHAO Y. (2014). Numerical simulation unsteady cloud cavitating flow with a filter-based density correction model. *Journal of Hydrodynamics*.

[28] Chapman M. S., Plesset R. B. (1971). Thermal effects in the free oscillation of gas bubbles. *Journal of Basic Engineering*.

[29] Plesset R. B., Chapman M. S. (1971). Collapse of an initially spherical vapour cavity in the neighbourhood of a solid boundary. *Journal of Fluid Mechanics*.

[30] Dular M., Coutier-Delgosha O. (2009). Numerical modelling of cavitation erosion. *International Journal for Numerical Methods in Fluids*.

[31] Blake J. R. (1988). The Kelvin impulse: application to cavitation bubble dynamics. *The Journal of the Australian Mathematical Society. Series B. Applied Mathematics*.

[32] Pereira F. (1997). *Prediction de l'erosion de cavitation: approche energetique, [Ph.D. thesis]*.

